# Biofluid-based staging of Alzheimer’s disease

**DOI:** 10.1007/s00401-025-02863-w

**Published:** 2025-03-17

**Authors:** Juan Lantero-Rodriguez, Laia Montoliu-Gaya, Nicholas J. Ashton, Ilaria Pola, Joseph Therriault, Nesrine Rahmouni, Wagner S. Brum, Stijn Servaes, Jenna Stevenson, Guglielmo Di Molfetta, Burak Arslan, Jesse Klostranec, Paolo Vitali, Maxime Montembeault, Serge Gauthier, Cecile Tissot, Arthur C. Macedo, Tharick A. Pascoal, Andreas Jeromin, Johan Gobom, Kaj Blennow, Henrik Zetterberg, Pedro Rosa-Neto, Andrea L. Benedet

**Affiliations:** 1 Department of Psychiatry and Neurochemistry, Institute of Neuroscience & Physiology, The Sahlgrenska Academy at the University of Gothenburg, Mölndal, Sweden; 2 Wallenberg Centre for Molecular and Translational Medicine, University of Gothenburg, Gothenburg, Sweden; 3 Department of Old Age Psychiatry, Maurice Wohl Clinical Neuroscience Institute, King’s College London, London, UK; 4 NIHR Biomedical Research Centre for Mental Health & Biomedical Research Unit for Dementia at South London & Maudsley NHS Foundation, London, UK; 5 Montreal Neurological Institute, Montreal, QC Canada; 6 Department of Neurology and Neurosurgery, McGill University, Montreal, QC Canada; 7 Graduate Program in Biological Sciences: Biochemistry, Universidade Federal Do Rio Grande Do Sul (UFRGS), Porto Alegre, Brazil; 8 Lawrence Berkeley National Laboratory, Berkeley, CA USA; 9 Department of Neurology and Psychiatry, University of Pittsburgh School of Medicine, Pittsburgh, USA; 10 ALZpath, Inc., Carlsbad, CA USA; 11 Clinical Neurochemistry Laboratory, Sahlgrenska University Hospital, Mölndal, Sweden; 12 Department of Neurodegenerative Disease, Queen Square Institute of Neurology, University College London, London, UK; 13 UK Dementia Research Institute, University College London, London, UK; 14 Hong Kong Center for Neurodegenerative Diseases, Hong Kong, China; 15 Wisconsin Alzheimer’s Disease Research Center, University of Wisconsin School of Medicine and Public Health, University of Wisconsin-Madison, Madison, WI USA

**Keywords:** Plasma, CSF, Staging, Alzheimer, Tau, p-Tau

## Abstract

Recently, conceptual systems for the in vivo staging of Alzheimer’s disease (AD) using fluid biomarkers have been suggested. Thus, it is important to assess whether available fluid biomarkers can successfully stage AD into clinically and biologically relevant categories. In the TRIAD cohort, we explored whether p-tau217, p-tau205 and NTA-tau (biomarkers of early, intermediate and late AD pathology, respectively) have potential for biofluid-based staging in cerebrospinal fluid (CSF; *n* = 219) and plasma (*n* = 150), and compared them in a paired CSF and plasma subset (*n* = 76). Our findings suggest a good concordance between biofluid staging and underlying pathology when classifying amyloid-positivity into three categories based on neurofibrillary pathology: minimal/non-existent (p-tau217 positive), early-to-intermediate (p-tau217 and p-tau205 positivity), and advanced tau tangle deposition (p-tau217, p-tau205 and NTA-tau positive), as indexed by tau-PET. Discordant cases accounted for 4.6% and 13.3% of all CSF and plasma measurements respectively (9.2% and 11.8% in paired samples). Notably, CSF- and plasma-based staging matched one another in 61.7% of the cases, while approximately 32% of the remaining participants were one to three biofluid stages higher in CSF as compared to plasma. Overall, these exploratory results suggest that biofluid staging of AD holds potential for offering valuable insights into underlying AD hallmarks and disease severity. However, its applicability beyond molecular characterization at research settings has yet to be demonstrated.

**Supplementary Information:**

The online version contains supplementary material available at 10.1007/s00401-025-02863-w.

## Introduction

Alzheimer’s disease (AD) is of great concern worldwide given its socioeconomic burden in a growing aging population [6, 8]. Presently, definitive diagnosis of AD is established upon post-mortem brain tissue examination confirming the presence of aggregated amyloid-β (Aβ) peptides into Aβ plaques and hyperphosphorylated tau protein into neurofibrillary tangles (NFTs) [9, 11]. Because these pathological hallmarks can be identified and monitored in living individuals, the National Institute on Aging and the Alzheimer's Associations (NIA-AA) Research Framework established that AD is a biological construct defined in vivo by fluid and imaging biomarkers. Based on the pathological process they measure, these biomarkers are classified into the so-called AT(N) system: Aβ pathology (A), tau pathology (T) and neurodegeneration (N) [11, 14].

However, the traditional classification of fluid phosphorylated tau (p-tau) as T biomarkers might be a slight oversimplification. First, the emergence of different p-tau species across the AD *continuum* differs from one another [2, 34]. In CSF and plasma, p-tau231 is the earliest abnormally emerging p-tau species [1, 34], and it is more strongly associated with Aβ pathology than tau pathology [31, 40]. P-tau217 emerges slightly after p-tau231 and shows a similar association with Aβ and tau pathology [15, 21, 22, 26, 31, 34, 40]. Finally, p-tau205, now available in a high throughput immunoassay platform [17], has been shown to increase later during the disease course compared with p-tau231, p-tau217 and p-tau181 [2, 3, 23, 24]. Importantly, p-tau205 levels in CSF and plasma displayed tighter association with neurofibrillary tau pathology than Aβ plaque pathology measured using PET or by neuropathological examination [3, 17, 24]. These relevant nuances have been also observed for total-tau (t-tau). Traditionally classified as N [14], the term total-tau alludes to the ability of these assays to measure all six central nervous system tau protein isoforms. However, t-tau immunoassay designs vary greatly based on the capture and detector antibodies used, which subsequently determines which pools of tau fragments are bound and measured [7, 33]. Consequently, some t-tau biomarkers appear to be, at least in the context of AD, more reflective of tau pathology than neurodegeneration [10, 18, 19]. A prime example of this is N-terminal tau fragments (NTA-tau), an immunoassay targeting tau fragments containing at least the N-terminal and the mid-region (irrespective of their phosphorylation state). In AD, NTA-tau levels in both plasma and CSF rise significantly during late disease stages, displaying a strong correlation with in vivo tangle pathology determined with tau-PET [18, 19, 33].

Echoing these and other findings, the AA recently published a revised criteria for the diagnosis and staging of AD in research settings [12]. The report introduced a conceptual staging scheme using fluid biomarkers: initial or A (abnormal Aβ1–42/40, p-tau181/Aβ1–42, accurate assays), early or B (other p-tau forms, e.g., p-tau205), intermediate (e.g., MTBR-243) and advanced stage (non-phosphorylated tau fragments) [12, 13]. However, these criteria highlight that the classification of p-tau205, MTBR-243 and non-phosphorylated tau as early, intermediate and advanced stage fluid markers (respectively) is currently preliminary, with further validation studies warranted [12]. Motivated by the AA proposal, we investigated a biofluid staging system defined by p-tau217, p-tau205 and NTA-tau as early, intermediate, and advanced stage fluid markers (respectively) of AD progression. There are two main reasons for including NTA-tau in the biofluid staging scheme: first, NTA-tau is measurable in CSF and blood [18, 19, 33] and second, NTA-tau increases during mid-to-late AD stages, is strongly associated with tau pathology, and can track tau deposition in AD [18, 19, 41]. Thereby, this staging system aligns more closely to the fluid biomarker scheme proposed by Therriault and colleagues [39].

In this explorative study, we investigated the idea of biofluid-based staging of AD using measurements of p-tau217, p-tau205 and NTA-tau in CSF and blood, and evaluate whether abnormalities in these biomarkers align with underlying pathological AD hallmarks. Additionally, we examined whether biofluid staging of AD using CSF and blood are interchangeable or if potentially relevant nuances exist between the two. To test this, we first classified individuals based on their CSF biomarker status into 4 groups, or CSF-based stages. Then, we evaluated how these CSF-based stages reflect clinical severity and tau-PET evidence of tau pathology. Next, we performed the same analysis but using plasma measures of these biomarkers. Finally, we compared the CSF and plasma staging classifications using a subset of participants who had these samples collected at the same time point.

## Methods

### Study participants

The cross-sectional samples presented here belong to the Translational Biomarkers of Aging and Dementia (TRIAD) cohort (McGill University, Montreal, Canada). Further details of the information gathered from participants can be found elsewhere (https://triad.tnl-mcgill.com/). All TRIAD participants here included underwent clinical evaluation, [^18^F]AZD4694 Aβ-PET, [^18^F]MK6240 tau-PET, magnetic resonance imaging (MRI), and lumbar puncture, for CSF collection (*n* = 219), or blood collection (*n* = 150). A subset of these participants had plasma and CSF samples collected at the same time point (*n* = 76). The average time to have all biomarkers acquired was of 196.4 (± 141) days.

Clinical diagnosis was established using an extensive neuropsychological assessment [27] that included Mini-Mental State Examination (MMSE), Clinical Dementia Rating (CDR) scores and the NIA-AA criteria. Cognitively unimpaired (CU) individuals presented no objective impairment, MMSE score of ≥ 26, and CDR score of 0. Participants with mild cognitive impaired (MCI) displayed objective cognitive impairment, MMSE score of ≥ 26, and CDR score of 0.5. AD dementia cases met the NIA-AA criteria for probable AD determined by a dementia specialist and presented MMSE score of < 26 and CDR of ≥ 0.5. Non-AD participants included dementia cases suspected of non-AD pathophysiology (AD biomarker negative based on Aβ-PET) and met clinical criteria for AD dementia, frontotemporal dementia (FTD, behavioural or semantic variant), progressive supranuclear palsy (PSP) or primary progressive aphasia (PPA); all with CDR > 0.5. Non-AD cases were diagnosed by a consensus panel of neurologists considering both clinical symptoms and brain images. Non-AD participants did not meet the criteria for other neurological or major neuropsychiatric disorder.

### CSF and plasma biomarker measurements

CSF levels of p-tau205 and NTA-tau were quantified in the TRIAD cohort with two in-house developed Simoa immunoassays. Both CSF assays were measured using a Simoa HD-X platform (Quanterix) at the Clinical Neurochemistry Laboratory, Sahlgrenska University Hospital, Mölndal, Sweden. Development and validation details for both immunoassays have been reported previously [17, 33]. In brief, CSF p-tau205 assay is comprised by a rabbit polyclonal antibody selective against phosphorylated tau at threonine 205 (ThermoFisher Scientific) and conjugated to paramagnetic beads, whereas biotinylated Tau12 (6-18aa, BioLegend) was used for detection. CSF NTA-tau assay uses HT7 antibody (159-163aa, ThermoFisher Scientific) conjugated to paramagnetic beads and biotinylated Tau12 (6-18aa, BioLegend) for detection. Prior to quantification using CSF p-tau205 and NTA-tau, samples were thawed at room temperature for 45 min, subsequently vortexed (30 s at 2000 rpm) and finally diluted using commercially available Tau2.0 assay diluent (Quanterix). CSF p-tau217 was measured using a commercially available kit (ALZpath) following the manufacturers recommendations and using a Simoa HD-X platform (Quanterix) at the Clinical Neurochemistry Laboratory, Sahlgrenska University Hospital, Mölndal, Sweden. The threshold to attribute a CSF biomarker status (positive/negative) was calculated as the mean biomarker concentrations of the CU Aβ negative group plus 2 SD (p-tau217 = 21.89 pg/mL; p-tau205 = 2.72 pg/mL; NTA-tau = 99.02 pg/mL).

Plasma levels of p-tau205 were quantified using a mass spectrometry (MS) method as previously described [24]. The use of MS instead of an immunoassay for measuring p-tau205 in plasma is due to the lack of an available plasma immunoassay for p-tau205. Plasma NTA-tau was quantified using the same assay specifications presented above for CSF measurements. Before quantification of plasma NTA-tau, samples were thawed at room temperature for 45 min, subsequently vortexed (30 s at 2000 rpm) and centrifuged (10 min at 4000 g), and finally diluted using commercially available Tau2.0 assay diluent (Quanterix). Plasma p-tau217 was measured using a commercially available kit (ALZpath) following the manufacturers recommendations. The threshold to attribute a CSF biomarker status (positive/negative) was calculated as the mean biomarker quantifications of the CU Aβ negative group plus 2 SD (p-tau217 = 0.56; p-tau205 = 0.000381 fm/mL; NTA-tau = 0.39 pg/mL).

All samples (CSF and plasma) were randomized and analysed blinded. Quality controls were included in both the immunoassays and MS method the repeatability and intermediate precision.

### Imaging processing

PET scans for Aβ and tau pathologies using [^18^F]AZD4694 and [^18^F]MK6240 were conducted 40–70 min and 90–110 min post-injection, respectively. Utilizing a Siemens High Resolution Research Tomograph (Siemens Medical Solutions, Knoxville, TN), imaging data were processed in conjunction with individual MRIs. The cerebellar grey matter and the inferior cerebellar grey matter served as reference regions for Aβ and tau-PET standard uptake value ratio (SUVR) calculation, the last also underwent skull-stripping before blurring to minimize spill-in from meningeal off-target binding. Aβ positivity was determined if the global neocortical [_18_F]AZD4694 SUVR equalled or exceeded 1.55 [36]. For tau-PET, a global index of tau pathology was obtained by calculating the average SUVR in the temporal meta-ROI region, with tau positivity defined as equal to or greater than 1.24 SUVR ([37]). Regional tau-PET was also quantified in the medial temporal and neocortical regions as previously published [35]. Additional cut-offs for "low" or "advanced" tau accumulation were established using 2.5 standard deviations of the mean medial temporal (≥ 1.03) and neocortical (≥ 1.06) SUVR of young participants, respectively. Participants were also categorized in PET-based Braak stages based on the topography of tau-PET abnormality, as described previously [28].

### Statistical analysis

The statistical analyses were conducted using the R software package (version 4.3.2), with a significance level set at *P* < 0.05 (two-sided). Demographic characteristics were described using the chi-square test for sex proportions and one-way ANOVA for age comparisons between groups. Fluid biomarker cut-offs were determined as the mean ± 2 SD of cognitively unimpaired Aβ negative participants: p-tau217 (CSF 21.89 pg/mL; plasma 0.56 pg/mL), p-tau205 (CSF 2.72 pg/mL; plasma 0.000381 fm/mL), NTA-tau (CSF 99.02 pg/mL; plasma 0.39 pg/mL) (thresholds are indicated in Supplementary Fig. S1 and S2). First, participants were categorized based on fluid biomarker stage as follows: stage-0 = p-tau217-/ p-tau205-/ NTA-tau-; stage-1 = p-tau217 + / p-tau205-/ NTA-tau-; stage-2 = p-tau217 + / p-tau205 + / NTA-tau-; stage-3 = p-tau217 + / p-tau205 + / NTA-tau + ; Discordant (D) = not fitting in the previous groups (Supplementary Fig. S3). In unpaired samples, discordant cases accounted for 4.6% and 13.3% of all CSF and plasma measurements respectively, whereas in paired samples accounted for 9.2% and 11.8%. Next, participants were also grouped, on different analyses, according to their clinical diagnosis, AT status, Braak stages, Aβ-PET status and tau-PET status in the medial and neocortical temporal regions.

Biomarker z-scores were calculated using the average values of CU Aβ-PET negative individuals as a reference. For parametric tests, fluid biomarkers were log-transformed as appropriate. Linear regression models compared biomarker levels across diagnostic groups while adjusting for age and sex. When comparing tracer uptake in relation to biofluid-based stages, linear regression was used to contrast a higher-level stage to its preceding stage only. To identify the optimal combination of biomarkers that best explained the variability in tau-PET load, we employed the “*Multi-Model-Inference*” R package. The function tests models with all possible combination of biomarkers and covariates (age and sex) and then ranks these models based on the second-order Akaike Information Criteria (AICc). The model with the lowest AICc indicates the best fit, with changes of less than two points suggesting a similar fit. In case of similar fitting, the models would be compared using ANOVA to select the most parsimonious. Next, to evaluate biomarker “trajectories” in relation to Aβ and tau-PET SUVR locally estimated scatterplot smoothing (LOESS) regression (span = 1) was used.

At the voxel level, the average Aβ- and tau-PET uptakes were calculated for each biofluid-based stage using MincTools.

## Results

### Demographics

A total of 219 participants were included in the CSF data analysis and 150 participants in the plasma data analysis. The median population age was 67.8 and 71.7 years in the CSF and plasma datasets, respectively. No statistical difference in sex distribution was found across groups, in both datasets, but cognitively unimpaired (CU) Aβ- (CU-) participants were younger than participants of other groups (Table 1; summary information regarding the subset of participants with both CSF and plasma data [*n* = 75] can be found in Supplementary Table S1). As expected, fluid and imaging biomarkers of Aβ and tau pathologies increased across the AD spectrum but were not found to be different across CU-, Aβ- participants with mild cognitive impairment (MCI-) and non-AD dementia (Non-AD) groups (Supplementary Figs. S1 and S2).

**Table 1:** Demographic and biomarker information of the CSF and plasma datasets

	CU-	CU+	MCI+	ADD	MCI-	Non-AD
CSF dataset	(*N* = 98)	(*N* = 24)	(*N* = 31)	(*N* = 31)	(*N* = 16)	(*N* = 19)
Sex, female	59 (60.2%)	15 (62.5%)	18 (58.1%)	17 (54.8%)	7 (43.8%)	12 (63.2%)
Age, years	57.5(21.7)	69.9(9.81)	71.5(5.61)	64.7(7.26)	70.4(10.1)	63.0(7.60)
p-tau217, pg/mL	9.04 (6.79–13.59)	32.5 (23.53–49.56)	53.3 (38.55–78.39)	69.0 (48.42–128.83)	10.9 (8.00–17.39)	9.49 (5.98–11.18)
p-tau205, pg/mL	1.61 (1.25–2.01)	2.82 (1.90–3.42)	3.75 (2.93–4.63)	4.30 (3.22–7.22)	1.98 (1.78–2.12)	1.50 (1.17–2.02)
NTA-tau, pg/mL	34.1 (24.84–56.54)	71.10 (45.52–95.59)	93.9 (68.35–120.93)	117 (79.28–198.93)	53.6 (43.93–77.05)	28.5 (22.85–37.11)
Med. Temp. tau-PET, SUVR	0.81(0.12)	1.15(0.40)	1.72(0.68)	2.23(0.55)	0.83(0.14)	0.81(0.16)
Neocort. tau-PET, SUVR	0.82(0.08)	0.92(0.30)	1.31(0.58)	2.41(0.98)	0.81(0.09)	0.81(0.12)
Aβ-PET, SUVR	1.27(0.09)	2.02(0.41)	2.41(0.49)	2.33(0.48)	1.34(0.10)	1.28(0.21)
Stage 0	87 (88.8%)	5 (20.8%)	3 (9.7%)	0 (0%)	13 (81.3%)	18 (94.7%)
Stage 1	1 (1.0%)	6 (25.0%)	4 (12.9%)	4 (12.9%)	0 (0%)	0 (0%)
Stage 2	0 (0%)	8 (33.3%)	11 (35.5%)	8 (25.8%)	0 (0%)	0 (0%)
Stage 3	3 (3.1%)	5 (20.8%)	13 (41.9%)	19 (61.3%)	1 (6.3%)	0 (0%)
Stage discordant	7 (7.1%)	0 (0%)	0 (0%)	0 (0%)	2 (12.5%)	1 (5.3%)

Plasma dataset	(*N* = 41)	(*N* = 28)	(*N* = 34)	(*N* = 28)	(*N* = 10)	(*N* = 9)
Sex, female	21 (51.2%)	20 (71.4%)	17 (50.0%)	22 (78.6%)	6 (60.0%)	6 (66.7%)
Age, years	64.8(18.7)	72.3(8.16)	73.3(5.40)	69.8(7.02)	71.6(4.91)	71.1(6.06)
p-tau217, pg/mL	0.25 (0.18–0.34)	0.68 (0.42–0.92)	0.94 (0.70–1.40)	1.58 (1.19–2.19)	0.20 (0.18–0.31)	0.34 (0.18–0.55)
p-tau205, fm/mL	0.00020 (0.00009–0.00025)	0.00032 (0.00024–0.00039)	0.00040 (0.00027–0.00056)	0.00073 (0.00054–0.00095)	0.00019 (0.00012–0.00058)	0.00025 (0.00021–0.00045)
NTA-tau, pg/mL	0.19 (0.13–0.26)	0.25 (0.15–0.33)	0.34 (0.20–0.49)	0.74 (0.49–0.89)	0.20 (0.18–0.28)	0.21 (0.17–0.43)
Aβ-PET, SUVR	1.28(0.10)	2.10(0.32)	2.40(0.43)	2.58(0.47)	1.31(0.099)	1.21(0.144)
Med. Temp. tau-PET, SUVR	0.84(0.15)	1.23(0.49)	1.83(0.71)	2.37(0.67)	0.862(0.14)	0.82(0.11)
Neocort. tau-PET, SUVR	0.84(0.082)	0.90(0.14)	1.37(0.60)	2.53(1.00)	0.80(0.11)	0.79(0.09)
Stage 0	33 (80.5%)	7 (25.0%)	5 (14.7%)	2 (7.1%)	7 (70.0%)	5 (55.6%)
Stage 1	2 (4.9%)	11 (39.3%)	7 (20.6%)	1 (3.6%)	0 (0%)	0 (0%)
Stage 2	1 (2.4%)	4 (14.3%)	8 (23.5%)	3 (10.7%)	0 (0%)	0 (0%)
Stage 3	1 (2.4%)	1 (3.6%)	10 (29.4%)	20 (71.4%)	1 (10.0%)	1 (11.1%)
Stage discordant	4 (9.8%)	5 (17.9%)	4 (11.8%)	2 (7.1%)	2 (20.0%)	3 (33.3%)

Data are presented as count (%) or mean (Standard Deviation, SD), except for the fluid biomarkers which were given in median (Q1-Q3)

+  Amyloid PET positive, − Amyloid PET negative, *Aβ* Amyloid, *ADD* Alzheimer’s disease dementia, *CSF* Cerebrospinal fluid, *CU* Cognitively unimpaired, *MCI* Mild cognitive impairment, *Med. Temp* Medial temporal, *Neocort.* Neocortical, *NTA-tau* N-terminal tau fragments, *PET* Positron emission tomography, *p-tau* Phosphorylated tau, *Q* quartile, *SUVR* Standard uptake value ratio

### CSF-based stages

**Fig. 1:**
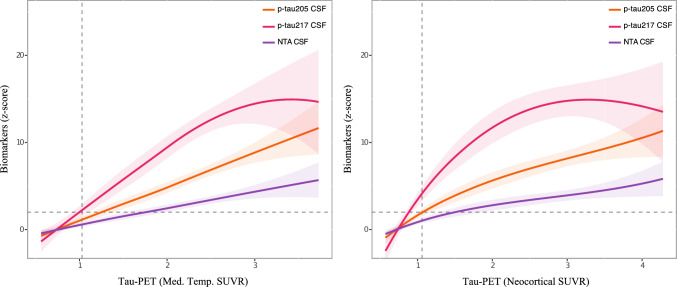
CSF tau biomarker trajectories in the TRIAD cohort across (**A**) medial temporal and (**B**) neocortical tau-PET SUVRs using a local weighted regression method (Loess curve). Changes in CSF biomarker levels are represented as Z-scores using tau-PET SUVRs as a proxy of AD pathology progression. Abnormal biomarker levels are determined as two standard deviations (SD) above the mean. Biomarker thresholds for positivity are indicated by the dashed lines

Supported by the current research framework which highlights that CSF biomarker abnormalities are more reflective of brain changes and occur prior to their plasmatic counterparts, we used the CSF data to infer the biomarker order. Using tau-PET uptake in the medial and neocortical temporal regions to proxy early and advanced tau pathology, respectively, we first evaluated which combination of biomarkers would generate a statistical model that best explained the variance in tau burden in these brain regions (Supplementary Table S2). The results indicated that combining the three biomarkers plus age best explained tau pathology load in these brain regions. Furthermore, considering that thresholds of biomarker abnormality were generated based on CU- (as detailed below), the level at which each biomarker becomes abnormal can also be suggestive of their order of “emergence”. For instance, by observing the biomarker distribution across groups, a p-tau217 cut-off could well separate CU- from CU+ participants (AUC = 0.97, 95% CI 0.82–0.99), while p-tau205 could only have similar performance at the MCI+ stage (AUC = 0.95, 95% CI 0.91–0.99), followed by NTA-tau that had similar discriminatory performance only at the AD dementia stage (AUC = 0.90, 95% CI 0.85–0.95; all AUC values reported in Supplementary Table S3). Using a locally estimated scatterplot smoothing (LOESS), we also modelled the cross-sectional pseudo-trajectories of the studied CSF tau biomarkers using tau-PET (medial temporal and neocortical SUVR) as a proxy of tau pathology progression in AD (Fig. 1; individual associations are presented on Supplementary Figure S4). CSF p-tau217, p-tau205, and NTA-tau levels increased at greater medial temporal and neocortical tracer uptake, supporting the feasibility of a biofluid-based staging of AD progression using these three markers.

**Fig. 2:**
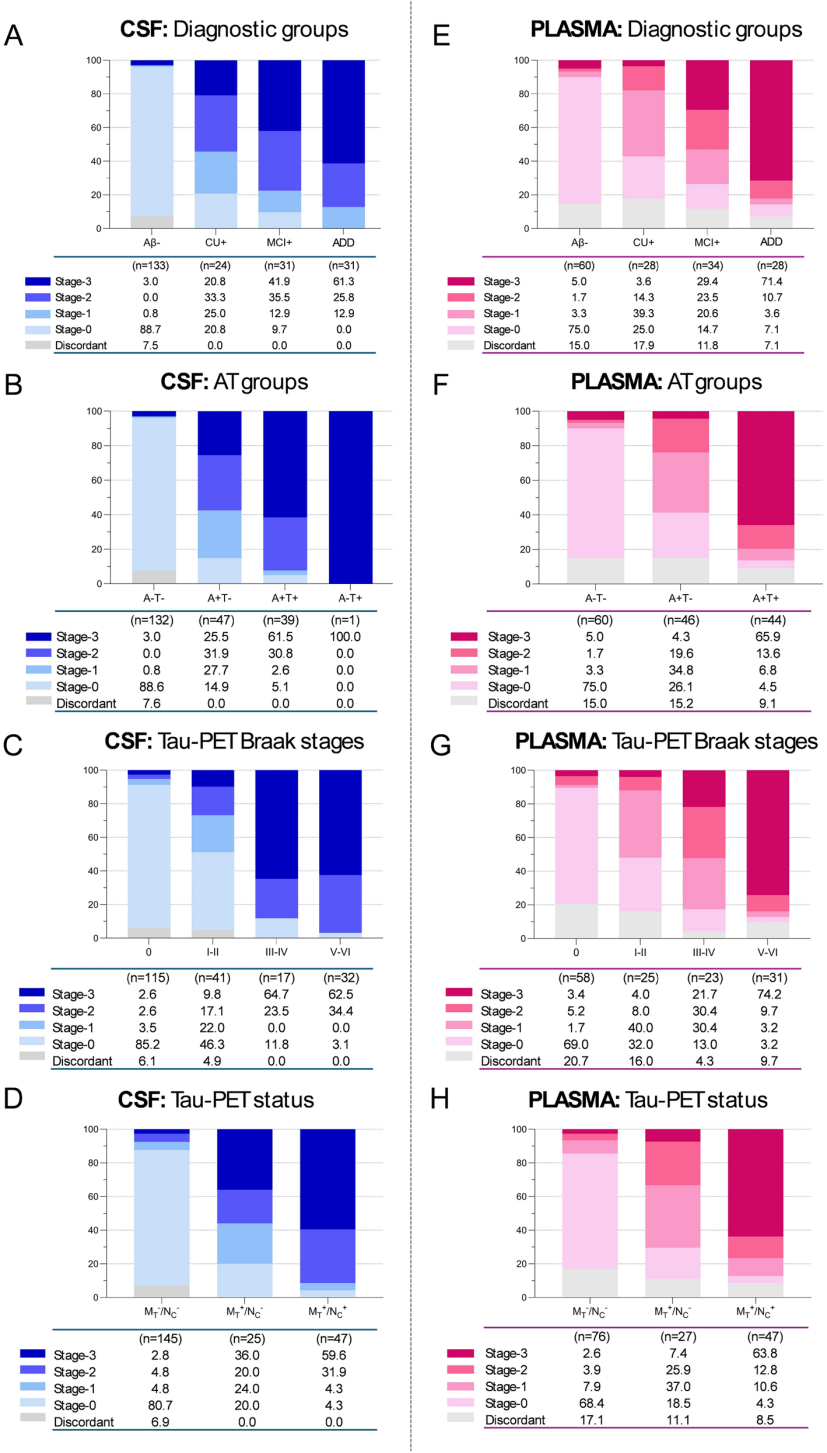
Biofluid-based staging across clinically relevant stratifications in non-paired CSF and plasma samples. Stacked bar charts represent the percentages of fluid stage-0, stage-1, stage-2 and stage-3 participants. In blue, CSF staging across (**A**) diagnostic groups, (**B**) AT groups, (**C**) tau-PET defined Braak stages, and (**D**) tau-PET status. In pink, plasma staging across (**E**) diagnostic groups, (**F**) AT groups, (**G**) tau-PET defined Braak stages, and (**H**) tau-PET status. Discordant cases with the biofluid staging criteria are shown in grey. Table shows the percentages and total number of individuals in each group

Next, we investigated CSF p-tau217, p-tau205 and NTA-tau across different clinically relevant stratifications in non-matched samples (Fig. 2A–D). For this purpose, we generated cut-offs for each biomarker using the CU- group (thresholds are indicated in Supplementary Fig. S1). Thus, using these three tau biomarkers and tracking their abnormalities throughout the disease *continuum*, the following biofluid-based stages were generated: stage-0 (negative for all three soluble tau markers), stage-1 (p-tau217 positive only), stage-2 (p-tau217 and p-tau205 positive, NTA-tau negative) and stage-3 (p-tau217, p-tau205 and NTA-tau positive). Cases in disagreement with the hypothesized sequence of biomarker abnormalities were considered discordant (see Table 1 and Methods). In CSF, Aβ- participants were mostly classified as stage-0 (88.7%), while CU+ cases included a similar proportion of each CSF biomarker stage. The shift towards double- and triple-positive cases became apparent in MCI+ and AD groups (Fig. 2A). A very similar pattern was observed when evaluating the proportion of CSF-based stages within AT groups, with the A+T- group mostly including fluid biomarker positive individuals at different CSF-based stages (85.1%) whereas the A+T+ group was almost fully comprised by CSF stage-2 (30.8%) and CSF stage-3 (61.5%) (Fig. 2B). When focusing on tau-PET outcomes, tau-PET Braak 0 group mainly consisted of stage-0 individuals (85.2%). Progressively, the number of fluid biomarker positive participants increased along with advancing tau-PET Braak stages, with the proportion in CSF stage-3 cases seemingly plateaued between tau-PET Braak III–IV and Braak V–VI (Fig. 2C). Finally, the Medial temporal tau (M_T_)^−^/Neocortical tau (N_C_)^−^ group was predominantly comprised by stage-0 cases (80.7%). Across M_T_^+^/N_C_^−^ and M_T_^+^/N_C_^+^ groups, an increasingly higher percentage in fluid biomarker positive cases was noticeable, particularly for those belonging to stage-2 and stage-3, which represented 56% and 91.5% of all cases in M_T_^+^/N_C_^−^ and M_T_^+^/N_C_^+^ groups, respectively (Fig. 2D).

### Plasma-based stages

We investigated whether the same biofluid-based staging could also be applied using plasma markers. For this, we used the same methodological approach for determining biofluid staging groups (thresholds are indicated in Supplementary Figure S2). In plasma, stage-0 represented 75% of all Aβ- cases. CU- participants were distributed mostly within plasma stages-0 (25%) and stage-1 (39.3%), while MCI+ individuals were almost evenly distributed across plasma stages-1, -2 and -3 (20.6%, 23.5% and 29.4%, respectively). AD dementia individuals comprised mostly plasma stage-3 participants (71.4%) (Fig. 2E). Across AT groups, A-T- included mainly stage-0 individuals (75.0%), A+T- relatively even percentages of plasma stage-0, -1 and -2 (19.6 to 34.8%), while the A+T+ group was dominated by stage-3 cases (65.9%) (Fig. 2F). As observed in the CSF analysis, the tau-PET Braak 0 group included primarily plasma stage-0 participants (69%), whereas the number of blood biomarker positive cases was increasingly present at more advanced tau-PET Braak stages (Fig. 2G). However, the increase in the percentage of plasma stage-2 and -3 cases across tau-PET Braak stages was less pronounced compared to that of CSF-based staging (Fig. 2G). Similarly, when tau-PET status was evaluated, M_T_^−^/N_C_^−^ group was again comprised mainly by plasma stage-0 cases (68.4%), whereas the increase in biomarker positive individuals across M_T_^+^/N_C_^−^ and M_T_^+^/N_C_^+^ groups was less pronounced in plasma when compared to CSF (Fig. 2H).

### Biofluid-based stages and in vivo pathology

**Fig. 3:**
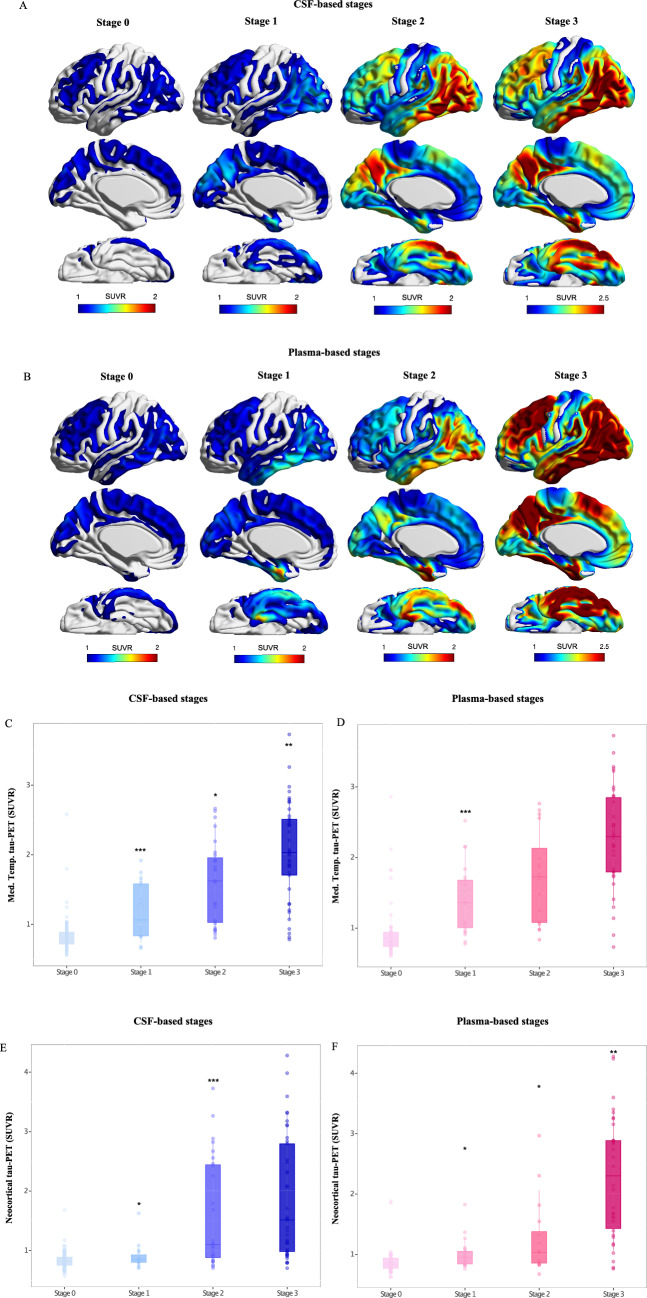
Average tau-PET uptake across the brain for each of the (**A**) CSF-based stages and (**B**) plasma-based stages. The boxplots depict the median (horizontal bar), 25th to 75th percentiles (hinges) and whiskers indicate 10th and 90th percentiles. Linear regression models contrasted a higher stage to its preceding stage, adjusting for age and sex. The * indicates significantly different tau-PET uptake in medial temporal tau-PET SUVR in (**C**) CSF- and (**D**) plasma-based stages, followed by similar comparisons in (**E–F**) neocortical tau-PET SUVR

We then investigated the extent of underlying neurofibrillary and plaque pathology, determined with PET, across CSF and plasma-based stages. In CSF stage-1 (Fig. 3A), a subtle degree of tau-PET binding was observed in inferior temporal regions. At CSF stage-2 (p-tau217 and p-tau205 positive), tau-PET retention was largely present in the upper temporal and parietal regions, and to a lesser extent in frontal cortex. The magnitude of tau-PET uptake in these regions was more overt in CSF stage-3 individuals. The plasma-based classification showed a similar pattern of regional tau-PET binding as observed for CSF-bases stages (Fig. 3B). We then compared the tau-PET tracer uptake across CSF- and plasma-based stages in medial temporal and neocortical regions as well as within tau-PET Braak regions. Both CSF and plasma stages followed a stepwise increase in tracer uptake in medial temporal regions (Fig. 3C, D) as well as in tau-PET Braak regions I–II (Supplementary Fig. S5 A-B), where each later stage had higher tau-PET SUVR than its preceding. When evaluating neocortical tau and tau-PET Braak III–IV regions, tau-PET SUVR was increased in CSF-based stage-1 and -2 compared with preceding CSF stage-0 (Fig. 3E). For plasma-based stages, tau-PET uptake in these same brain regions increased across stages (the most prominent increased occurred in plasma stage-3; Fig. 3F). Finally, tau-PET SUVR in regions corresponding to tau-PET Braak stages V–VI was only increased at biofluid-based stages-2 and -3 (in plasma, the increase was especially prominent in stage-3; Supplementary Figure S5 E–F). To complement this analysis, similarly to tau-PET, we also compared Aβ-PET SUVR levels between biofluid-based stages. For both CSF and plasma, a significant difference in Aβ load was found between stage-1 and -0, but no differences were found between stage-1 and -2, or -2 and -3 (Supplementary Fig. S6).

### The CSF- and plasma-based staging agreement

**Fig. 4:**
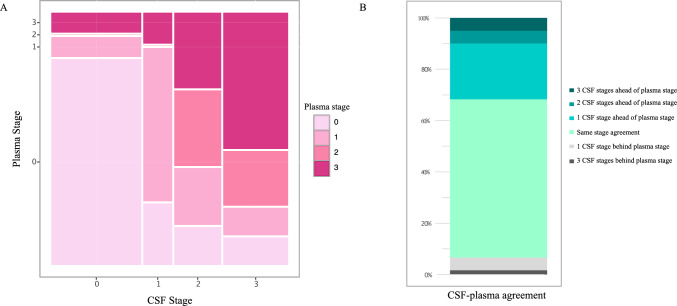
Agreement between CSF- and plasma-based stages in the matched dataset. The mosaic plot shows in (**A**) the proportions of individuals classified at each biofluid-based stage. The stacked bar chart (**B**) shows the proportion of individuals with some level of agreement between CSF and plasma stages in green shades whilst the grey shades indicate disagreement between these biofluid-based stages in which the plasma is more “advanced” as compared to the CSF classification

Both biofluid-based staging systems appeared to perform relatively similar on extreme classification groups, that is Aβ- vs AD, A-T- vs A+T+ , tau-PET Braak 0 vs V-VI, and M_T_^−^/N_C_^−^ vs M_T_^+^/N_C_^+^ (Fig. 2). However, because previous analyses were performed in two different sets of participants, questions remained to be answered: are plasma-based stages translatable to CSF-based stages? To which degree do they correspond to each other? Thus, we evaluated a subset of participants (*n* = 76) who had CSF and plasma paired samples collected at the same time-point. When first examining stacked bar plots across clinical and imaging-based classifications (Supplementary Fig. S7), the pattern observed on this subsample agrees with what observed in the previous analyses: the CSF staging system displays a more stepwise distribution of biomarker positivity across the different clinical and imaging-based classifications (Supplementary Fig. S7A–D), whereas the plasma staging criteria shows a more abrupt or sudden presence of triple positive or stage-3 participants in advanced disease groups, specifically AD, A+T+, tau-PET Braak V–VI, and M_T_^+^/N_C_^+^ (Supplementary Figure S7E-H). At first, this suggested a good agreement between the classifiers, at least between clinical and imaging-based extreme groups. However, when analysing the data more closely, at the individual level, the degree of same-level concordance between CSF and plasma stages was approximately 61.7% (37 out of 60 of concordant cases) (Fig. 4). Interestingly, in 31.2% percent of the cases (*n* = 19), the CSF-based classification placed individuals in higher level groups as compared to the plasma-based classification (21.75 +1 stage, 5% +2 stage, and 5% +3 stage), further supporting the framework that tau biomarkers first become “abnormal” in CSF and later in plasma. This is especially evident when examining participants belonging to “intermediate” classifications, such as MCI+ , A+T-, tau-PET Braak III–IV and M_T_^+^/N_C_^−^ (Supplementary Fig. S7). These groups are largely comprised by CSF defined stage-3 participants, whereas when using the plasma-based staging most individuals are classified as plasma stage-1 and stage-2. Importantly, we observed that the CSF staging classification across tau-PET Braak stages followed a stepwise increase in the number of CSF stage-3 cases.

This difference was further evidenced when levels of fluid and tau-PET biomarkers were plotted along the CSF- and plasma-based stages, side by side (Supplementary Figure S8). Across CSF stages, the bulk of the increase in medial temporal and neocortical tau-PET was located between CSF stages-1 and -3. Using this classification, CSF p-tau205 increase in parallel with tau-PET uptake, especially in medial temporal region. When using plasma-based stages, most of the increase in neocortical tau SUVRs occurred between plasma stage-2 and -3, whereas the medial temporal tau SUVRs increased progressively across all plasma-based stages. Importantly, when comparing the cross-sectional pseudo-trajectories of the fluid biomarkers using both classification systems, it became apparent that the same tau species did not increase in parallel to one another in the two matrices; in other words, CSF and plasma tau fluid measurements were not interchangeable for defining an individual’s biofluid-based stage.

## Discussion

The present study reports an explorative evaluation of a biofluid-based staging system for CSF and blood using three different tau fluid markers (p-tau217, p-tau205 and NTA-tau) as representative markers of early, intermediate and advanced AD, respectively [12, 39]. Despite the cross-sectional nature of the study, CSF data suggested a stepwise increase in the selected tau biomarkers following the severity of AD pathology as observed with tau-PET. Following this, we generated cut-offs for each biomarker and defined four stages. Finally, we determined the presence of tau biomarkers abnormalities in CSF and plasma across stratifications of clinical significance, as well as their concordance in a subset of paired samples. Our main findings indicate that (i) fluid tau biomarker abnormalities might reflect the underlying extent of tau neurofibrillary pathology, (ii) CSF and plasma-based staging may provide valuable information regarding the biomarker phenotype of AD patients, and (iii) biofluid stages are not interchangeable between CSF and plasma in all individuals.

Previous studies suggest that sequential abnormalities in fluid measures of p-tau217, p-tau205 and NTA-tau are reflective of AD progression [2, 3, 10, 17–19, 24, 30, 39]. Therefore, we started by evaluating this in CSF, using medial temporal and neocortical tau pathology determined with PET as a proxy of disease progression and severity. The use of tau-PET was motivated by the fact that postmortem Braak stages have been shown to correlate with antemortem disease progression and cognitive decline [25, 32]. Our findings corroborate that fluid biomarker abnormalities are more commonly found at advanced disease stages, supporting the concept of a staging system of AD using these three biomarkers [39]. We also investigated how our biofluid staging system overlaid with clinically relevant stratifications (clinical diagnosis, AT groups, tau-PET status, and tau-PET Braak stages). The main difference between CSF and plasma-based staging was observed in intermediate groups (that is MCI+ , A+T-, tau-PET Braak III–IV and M_T_^+^/N_C_^−^), which were largely comprised by CSF stage-2 and -3, but plasma stage-1 and -2. At first glance, a delay could be hypothesized between plasma and CSF. However, this could only be evaluated in matched plasma and CSF samples. In the paired subset, the percentage of CSF stage-3 individuals increased across the classification criteria whereas in plasma, stage-3 appeared to be a more prominent biomarker phenotype of advanced disease groups (AD, A+T+ , tau-PET Braak V–VI, and M_T_^+^/N_C_^+^). In cases of disagreement between CSF and plasma, CSF was generally one fluid stage higher compared to plasma, which aligns with previously reported biomarker trajectories in both matrices [34, 38]. Therefore, how does the observed asynchrony between CSF and plasma staging impact the way in which they reflect AD progression? Both globally and at the voxel level, CSF and plasma-based stages showed the expected Aβ-PET uptake levels: minimal tracer binding at stage-0, extensive binding at stage-1 and overt binding at stages-2 and -3. Regarding regional tau-PET analysis, CSF- and plasma-based stages enabled the classification Aβ-PET positivity into three distinct tau pathology groups: minimal or non-existent, early-to-intermediate, and late in vivo tau deposition. Notably, the regional tau-PET uptake across biofluid staging groups was concordant with neurofibrillary pathology progression in AD [27], which might suggest that these classification schemes could be insightful in certain contexts, such as research settings. When we further investigated this in terms of global tau-PET uptake across staging groups, the unpaired and paired data sets rendered similar findings. Despite the cross-sectional nature of the data, medial temporal tau-PET uptake was higher in advanced CSF and plasma stages, suggesting that biofluid staging systems could provide valuable information in the absence of imaging alternatives, perhaps even tracking the progressive accumulation of tau pathology early in the disease course. Second, neocortical tau-PET uptake was only and similarly increased in CSF stage-2 and -3, whereas in plasma high tracer uptake was prominently observed in plasma stage-3 (more limited in plasma stage-2). This suggests that if using the proposed biofluid staging, someone classified as CSF stage-2/-3 or plasma stage-3 is likely to have neocortical tau-PET uptake above the positivity threshold. To further assess these important nuances, we also analysed the tau tracer uptake in CSF and plasma stages but this time stratifying by tau-PET Braak stages. In tau-PET Braak I–II, tracer uptake was higher in advanced CSF and plasma stages. In tau-PET Braak III–IV and V–VI, SUVRs were only increased in CSF stages-2/-3, whereas in plasma the bulk of increase occurred a stage-3. Altogether, this strengthens the idea of CSF stage-2/-3 or plasma stage-3 being indicative of high global tau-PET uptake, advanced medial temporal tangle deposition, positive neocortical tau, and tau-PET Braak III to VI status, whereas being CSF stage-1 or plasma stage-1/-2 likely signifies low global tau-PET uptake, early medial temporal tau accumulation, negative neocortical tau, and tau-PET Braak I–II status. Moreover, these findings align well with the natural history of AD where p-tau217 increases prior to p-tau205 and tau-PET (which increase approximately in parallel), and NTA-tau emerging after [2, 15, 17–20, 38]. Finally, we corroborate previous studies which showed that p-tau205 positivity is an important milestone in AD progression as a biomarker of tau pathology [3, 17, 24], but most importantly, our results suggest that the underlying extend of tau deposition might be staged by further stratifying p-tau205 positive cases using NTA-tau, a biomarker of mid-to-late tau pathology in AD [18, 19].

The advent of several disease-modifying trials in AD has highlighted the importance of staging AD rather than merely identifying whether AD pathological changes are present or absent. In this context, biofluid-based systems for staging AD pose great potential as cost-effective tools in determining eligibility for clinical trials, assessing the success or failure of novel drugs, and help in guiding clinical care. In the present study, we provide evidence suggesting the feasibility of biofluid staging framework, which successfully stratifies the AD spectrum, into different degrees of tau pathology deposition. However, it is important to emphasize that these findings are exploratory, and the concept of fluid biomarker staging (using the studied biomarkers or others) will require further research and rigorous validation to fully crystallize. Nonetheless, our results suggest potential applications in the context of clinical trials. Therapeutic interventions aiming at early AD stages (*e.g.*, presence of Aβ pathology but lack of over tau pathology) would include biofluid-based stage-1 participants (p-tau217 positive), whereas an intervention focusing on individuals with Aβ pathology and initial signs of tau pathology would include biofluid-based stage-2 cases (p-tau217 and p-tau205 positive, NTA-tau negative). Furthermore, the studied staging scheme shows a good alignment with underlying neurofibrillary pathology [27], suggesting it may hold potential as a useful tool in the absence of imaging biomarkers when evaluating disease progression in clinical settings, as well as for assessing if novel therapeutic compounds successfully tackle or decelerate AD progression.

Another contribution of this study is investigating how the different emergence of CSF and blood biomarkers counterparts may impact the ability of staging systems to reflect underlying AD neuropathology. A previous study using machine-learning techniques and mass spectrometry measurements investigated a biological staging model for AD using CSF biomarkers [30], supporting the framework proposed by the AA. Here, we demonstrate that a model based on dichotomizing patients in positive and negative for each biomarker—though using fewer markers than the other approach—remains insightful. Additionally, we expanded our CSF findings using this model by including plasma samples, as well as a subset of paired CSF and plasma samples. However, while our data suggests that staging AD using plasma biomarkers is possible, this approach is likely to be less accurate than a CSF-based staging system due to inherent limitations associated with blood measurements. However, with advancements in technology and the development of more sensitive assays, plasma biomarkers may achieve accuracy comparable to CSF biomarkers, as recently reported [4]. Notably, our findings suggest that the consequence of the asynchronous increase of fluid biomarkers might be a lack of full alignment between CSF and plasma-based staging. Despite this, it should be noted that both staging systems reasonably stratified Aβ pathology positivity into minimal or non-existent tau pathology, and early-to-intermediate and advanced tau accumulation. Finally, we also identified some discordant cases which did not follow the expected agreement of tau biomarker abnormalities, and as could be anticipated, these were more common across plasma than CSF. CSF is a simpler biofluid, making measurements less susceptible to analytical interference with other molecules in the matrix. Secondly, brain-derived molecules in blood are vulnerable to kidney clearance, liver metabolism and proteases. Finally, tau biomarkers are also expressed in non-cerebral tissue which can influence their plasma levels. Beyond this, biofluid staging may also be susceptible to analytical variation as well as biological variation (within- and between-subject biological variation), inherently present in soluble biomarker measurements, even in healthy individuals [5]. Therefore, if a fluid biomarker staging system is to be implemented in clinical settings or clinical trials, it must be preceded by thorough investigations into the variability of biofluid measurements.

This study presents some limitations. First, converting continuous biomarker quantifications into categorical classifications (that is bio-fluid stages) always leads to some inherent loss of information. Additionally, while the selection of a specific metric for cutoffs intends here to facilitate an exploratory dichotomization process (especially when considering the cohort size), future studies in larger cohorts containing more individuals and at more advanced disease stages should investigate in more detail participants at intermediate biomarker ranges. Predictably, if a fluid staging is ever adopted, it will be essential to carefully interpret biomarker readings near established thresholds, recognizing that biomarkers exist on a continuous spectrum but are artificially dichotomized into binary categories [12]. Second, the inclusion of Aβ42/40 would have been desirable. The exclusion of this biomarker in the present study was drove by three main reasons. (i) Aβ42/40 measurements were only available for a subset of individuals in plasma. As a result, we could not compare fluid staging between CSF and plasma datasets, nor assess paired datasets. (ii) The number of participants who were positive for CSF Aβ42/40 but negative for CSF p-tau and tau biomarkers was very small and would not allow us to make reasonable inferences with the data. To maintain a straightforward and interpretable exploratory staging approach, we chose to not create this subgroup. (iii) Current methods for plasma Aβ42/40 quantification exhibit significant overlap between Aβ-positive and Aβ-negative groups [16, 29]. Third, a validation cohort would have further strengthened our findings; however, we are currently unaware of other cohorts with available paired CSF and plasma samples, including Aβ and tau-PET, as well as soluble measures of p-tau217, p-tau205 and NTA-tau. Furthermore, due to the cross-sectional nature of this study, future studies are warranted to confirm the proposed sequential biomarker changes and biofluid-based staging longitudinally. Finally, we aimed to explore an immunoassay-based staging of AD, but unfortunately, while p-tau217 and NTA-tau could be measured in CSF and blood using the same analytical platform, this was not possible for plasma p-tau205, because the only available method was IP-MS.

In conclusion, this is the first study to investigate biofluid-based staging both in CSF and plasma. While the present results may provide insights on the feasibility of biofluid staging of AD, the establishment of fluid biomarker staging beyond a research tool still needs to undergo further investigation and extensive validation. Nevertheless, the studied fluid biomarker stages, both in CSF and plasma, showed a good degree of concordance with underlying AD hallmarks and a relevant stratification of the AD *continuum* into minimal or non-existent, early-to-mid, and advanced tau pathology determined with tau-PET. Moreover, this study highlights an asynchrony on biomarker emergence in CSF and plasma, and its potential impact on staging, which should be further investigated and considered when evaluating individuals for diagnosis, disease monitoring and treatment. Overall, our findings suggest that biofluid staging of AD might be one day a feasible assessment beyond research, with potential as a cost-effective tool when estimating disease severity, assessing patient prognosis, and determining eligibility for enrolment in clinical trials.

## Supplementary Information

Below is the link to the electronic supplementary material.

Supplementary file1 (DOCX 24274 KB)

## Data Availability

The present study includes no data deposited in external repositories. Bulk Anonymized data can be shared upon reasonable request from a qualified academic investigator for the sole purpose of replicating procedures and results presented in the article, providing data transfer is in agreement with EU legislation and decisions by the institutional review board of each participating research centre.

## Code availability

This study does not use customized algorithms or software.
