# Supplementary material for: Biofluid-based staging of Alzheimer’s disease
Source: Acta Neuropathol. 2025 Mar 17;149(1):27. doi: 10.1007/s00401-025-02863-w (PMC11913990; doi:10.1007/s00401-025-02863-w)
Supplement: Supplementary file 1 — Supplementary file1 (DOCX 24274 KB) [file 401_2025_2863_MOESM1_ESM.docx]

**TITLE: BIOFLUID-BASED STAGING OF ALZHEIMER’S DISEASE**

Juan Lantero-Rodriguez PhD^1*^, Laia Montoliu-Gaya PhD^1*^, Nicholas J. Ashton PhD^1,2,3,4^, Ilaria Pola MSc^1^, Joseph Therriault PhD^5,6^, Nesrine Rahmouni MSc^5,6^, Wagner S. Brum^1,7^, Stijn Servaes PhD^5,6^, Jenna Stevenson BSc^5,6^, Guglielmo Di Molfetta MSc^1^, Burak Arslan MD^1^, Jesse Klostranec MD PhD^5,6^_,_ Paolo Vitali MD PhD^5,6^_,_ Maxime Montembeault PhD^5,6^, Serge Gauthier MD PhD^5,6^_,_ Cecile Tissot PhD ^5,6,8^, Arthur C. Macedo MD^6,7^, Tharick A. Pascoal MD PhD^9^, Andreas Jeromin PhD^10^, Johan Gobon PhD^1^, Kaj Blennow MD PhD^1,11^, Henrik Zetterberg MD PhD ^1,11-15^, Pedro Rosa-Neto MD PhD^6,7^** and Andrea L. Benedet PhD^1^**

**Affiliations:**

^1^ Department of Psychiatry and Neurochemistry, Institute of Neuroscience & Physiology, The Sahlgrenska Academy at the University of Gothenburg, Mölndal, Sweden

^2^ Wallenberg Centre for Molecular and Translational Medicine, University of Gothenburg, Gothenburg, Sweden

^3^ Department of Old Age Psychiatry, Maurice Wohl Clinical Neuroscience Institute, King’s College London, London, UK

^4^ NIHR Biomedical Research Centre for Mental Health & Biomedical Research Unit for Dementia at South London & Maudsley NHS Foundation, London, UK

^5^ Montreal Neurological Institute, Montreal, QC, Canada

^6^  Department of Neurology and Neurosurgery, McGill University, Montreal, QC, Canada

^7^ Graduate Program in Biological Sciences: Biochemistry, Universidade Federal Do Rio Grande Do Sul (UFRGS), Porto Alegre, Brazil.

^8^ Lawrence Berkeley National Laboratory, Berkeley, CA, USA.

^9^ Department of Neurology and Psychiatry, University of Pittsburgh School of Medicine, Pittsburgh, USA

^10^ ALZpath, Inc., Carlsbad, California, USA.

^11^ Clinical Neurochemistry Laboratory, Sahlgrenska University Hospital, Mölndal, Sweden

^12^ Department of Neurodegenerative Disease, Queen Square Institute of Neurology, University College London, London, UK

^13^ UK Dementia Research Institute, University College London, London, UK

^14^ Hong Kong Center for Neurodegenerative Diseases, Hong Kong, China

^15^ Wisconsin Alzheimer’s Disease Research Center, University of Wisconsin School of Medicine and Public Health, University of Wisconsin-Madison, Madison, WI, USA

Contributed equally as first authors*

These authors jointly supervised this work: **

**TABLE OF CONTENTS**

**1. SUPPLEMENTARY TABLES**

**Table S1.** Demographics and biomarker information of the sample subset with paired CSF and plasma data.

**Table S2.** Comparison between models explaining the variance in tau burden.

**Table S3.** AUROC.

**2. SUPPLEMENTARY FIGURES**

**Figure S1.** CSF tau biomarkers across disease groups.

**Figure S2.** Plasma tau biomarkers across disease groups.

**Figure S3.** **Scatterplots showing the distribution of CSF and plasma tau biomarkers across fluid stages.**

**Figure S4.** **CSF tau biomarkers across tau PET load.**

**Figure S5.** Tau-PET tracer uptake across CSF and plasma stages in different Braak regions (unpaired samples).

**Figure S6.** Regional Aβ-PET uptake across CSF and plasma stages (unpaired samples).

**Figure S7.** Fluid-based staging across clinically relevant stratifications in paired CSF and plasma samples

**Figure S8.** Tau-PET tracer uptake across CSF and plasma stages in different Braak regions (paired samples).

**1. SUPPLEMENTARY TABLES**

**Table S1.** Demographics and biomarker information of the sample subset with paired CSF and plasma data.

|  | CU- (N=17) | CU+ (N=17) | MCI+ (N=18) | ADD (N=16) | MCI- (N=5) | Non-AD (N=3) |
| --- | --- | --- | --- | --- | --- | --- |
| Sex, female | 9 (52.9%) | 11 (64.7%) | 9 (50.0%) | 10 (62.5%) | 2 (40.0%) | 2 (66.7%) |
| Age, years | 72.6(5.31) | 72.2(5.03) | 73.1(5.27) | 66.7(7.00) | 73.0(4.49) | 72.5(4.75) |
| Med. Temp. tau-PET, SUVR | 0.82(0.12) | 1.24(0.49) | 1.77(0.71) | 2.16(0.68) | 0.83(0.13) | 0.85(0.13) |
| Neocort. tau-PET, SUVR | 0.82(0.08) | 0.91(0.15) | 1.31(0.58) | 2.33(1.11) | 0.77(0.10) | 0.80(0.10) |
| Aβ-PET, SUVR | 1.30(0.07) | 2.10(0.39) | 2.49(0.47) | 2.49(0.36) | 1.32(0.09) | 1.22(0.03) |
| **CSF** |  |  |  |  |  |  |
| p-tau217, pg/mL | 8.19  (6.50-10.53) | 30.6  (23.14–40.43) | 46.2  (30.23–75.69) | 69.9  (49.79–98.96) | 12.6  (7.54–19.63) | 12.5  (8.58–12.68) |
| p-tau205, pg/mL | 1.65  (1.51–1.92) | 2.45  (2.00–3.34) | 3.70  (2.99–4.89) | 4.26  (2.99–7.16) | 2.01  (1.79–2.28) | 1.43  (1.16–1.61) |
| NTA-tau, pg/mL | 43.1  (31.94–47.67) | 69.2  (55.39–103.13) | 86.6  (50.79–115.36) | 117  (83.17–181.43) | 72.5  (44.54–93.97) | 21.7  (20.67–29.50) |
| Stage 0 | 15 (88.2%) | 1 (5.9%) | 1 (5.6%) | 0 (0%) | 2 (40.0%) | 3 (100%) |
| Stage 1 | 1 (5.9%) | 6 (35.3%) | 1 (5.6%) | 0 (0%) | 0 (0%) | 0 (0%) |
| Stage 2 | 0 (0%) | 3 (17.6%) | 3 (16.7%) | 2 (12.5%) | 0 (0%) | 0 (0%) |
| Stage 3 | 0 (0%) | 6 (35.3%) | 12 (66.7%) | 13 (81.3%) | 0 (0%) | 0 (0%) |
| Stage Discordant | 1 (5.9%) | 1 (5.9%) | 1 (5.6%) | 1 (6.3%) | 3 (60.0%) | 0 (0%) |
| **Plasma** |  |  |  |  |  |  |
| p-tau217, pg/mL | 0.25  (0.22–0.34) | 0.59  (0.36–0.71) | 1.06  (0.75–1.36) | 1.38  (0.95–1.85) | 0.20  (0.21–0.29) | 0.54  (0.36–0.55) |
| p-tau205, fm/mL | 0.00020  (0.00009–0.00023) | 0.00032  (0.00023–0.00037) | 0.00042  (0.00037–0.00057) | 0.00062  (0.00041–0.00088) | 0.00017  (0.00010–0.00067) | 0.00025  (0.00018–0.00077) |
| NTA-tau, pg/mL | 0.19  (0.16–0.25) | 0.24  (0.15–0.27) | 0.36  (0.26–0.49) | 0.68  (0.47–0.86) | 0.20  (0.16–0.28) | 0.54  (0.33–1.09) |
| Stage 0 | 15 (88.2%) | 6 (35.3%) | 0 (0%) | 2 (12.5%) | 5 (100%) | 1 (33.3%) |
| Stage 1 | 1 (5.9%) | 7 (41.2%) | 2 (11.1%) | 1 (6.3%) | 0 (0%) | 0 (0%) |
| Stage 2 | 0 (0%) | 2 (11.8%) | 7 (38.9%) | 0 (0%) | 0 (0%) | 0 (0%) |
| Stage 3 | 0 (0%) | 0 (0%) | 7 (38.9%) | 11 (68.8%) | 0 (0%) | 0 (0%) |
| Stage discordant | 1 (5.9%) | 2 (11.8%) | 2 (11.1%) | 2 (12.5%) | 0 (0%) | 2 (66.7%) |

Data are presented as count (%) or mean (Standard Deviation, SD), except for the fluid biomarkers which were given in median (Q1-Q3).

*Abbreviations: +, Amyloid PET positive; -, Amyloid PET negative; Aβ, Amyloid; ADD, Alzheimer’s disease dementia; CU, Cognitively unimpaired; MCI, Mild cognitive impairment; Med. Temp, Medial temporal; Neocort., Neocortical; NTA-tau, N-terminal tau fragments; PET, Positron emission tomography; p-tau, Phosphorylated tau; Q, quartile; SUVR, Standard uptake value ratio.*

**Table S2.** Comparison between models explaining the variance in tau burden.

|  | **Age** | **p-tau217** | **p-tau205** | **NTA-tau** | **Sex** | **df** | **Log Lik.** | **AICc** | **Delta** | |
| --- | --- | --- | --- | --- | --- | --- | --- | --- | --- | --- |
| Med. Temp. tau-PET SUVR | | | | | | | | | |  |
| 1* | -0.004 | 0.203 | 0.444 | -0.159 |  | 6 | -110.21 | 232.8 | 0 | |
| 2 | -0.004 | 0.203 | 0.444 | -0.159 | + | 7 | -110.20 | 234.9 | 2.1 | |
| 3 |  | 0.199 | 0.440 | -0.174 |  | 5 | -112.77 | 235.8 | 3.01 | |
| 4 |  | 0.199 | 0.440 | -0.174 | + | 6 | -112.75 | 237.9 | 5.08 | |
|  |  |  |  |  |  |  |  |  |  | |
| Neocort. tau-PET SUVR | | | | | | | | | |  |
| 1 | -0.009 | 0.121 | 0.634 | -0.201 |  | 6 | -161.809 | 336.0 | 0 | |
| 2* | -0.009 | 0.120 | 0.635 | -0.200 | + | 7 | -161.621 | 337.8 | 1.76 | |
| 3 | -0.009 |  | 0.807 | -0.178 |  | 5 | -164.364 | 339.0 | 3 | |
| 4 | -0.009 |  | 0.807 | -0.177 | + | 6 | -164.128 | 340.7 | 4.64 | |

Biomarkers were log2 transformed when included in the model.

*Abbreviations: AICc, second-order Akffaike information criterion; df, degrees of freedom; Log Lik., Log likelihood ratio; Med. Temp, Medial temporal; Neocort., Neocortical; NTA-tau, N-terminal tau fragments; PET, Positron emission tomography; p-tau, Phosphorylated tau; SUVR, Standard uptake value ratio.*

**Table S3.** AUROC

|  | **AUROC (95% CI)** | | |
| --- | --- | --- | --- |
|  | **p-tau217** | **p-tau205** | **NTA-tau** |
| CU+ | 0.90 (0.82-0.99) | 0.80 (0.68-0.91) | 0.75 (0.63-0.86) |
| MCI+ | 0.97 (0.95-1.00) | 0.95 (0.91-0.99) | 0.84 (0.76-0.92) |
| ADD | 0.99 (0.98-1.00) | 0.99 (0.96-1.00) | 0.90 (0.85-0.95) |

All the analyses contrasted the above-mentioned groups against CU- individuals.

*Abbreviations: +, Amyloid PET positive; ADD, Alzheimer’s disease dementia; AUROC, Area under the receiver operating characteristic curve; CI, Confidence interval; CU, Cognitively unimpaired; MCI, Mild cognitive impairment; NTA-tau, N-terminal tau fragments; p-tau, Phosphorylated tau.*

**2. SUPPLEMENTARY FIGURES**

**
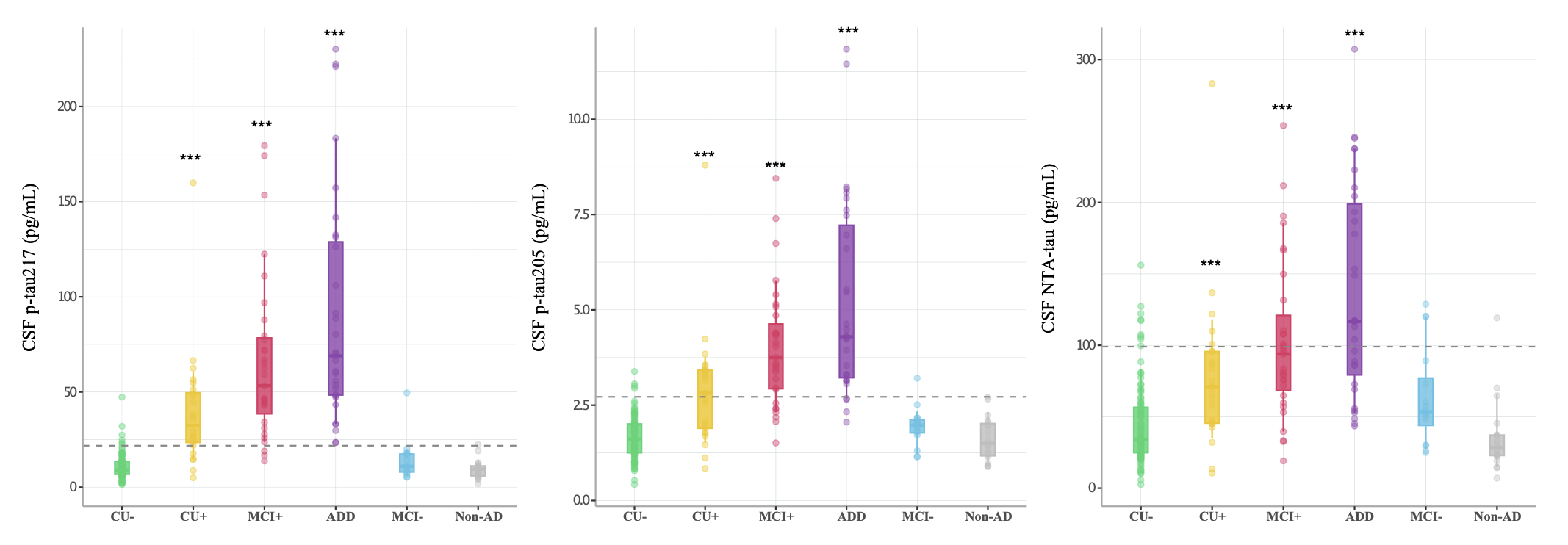
**

**Figure S1.** **CSF tau biomarkers across disease groups.** The boxplots depict the median (horizontal bar), 25th to 75th percentiles (hinges) and whiskers indicate 10th and 90th percentiles. Group comparisons were computed with linear regression models adjusting for age and sex. The * indicates, for each biomarker, the groups that are significantly different from CU- group (**P*<0.05, ***P*<0.01,

****P*<0.001). The dashed line indicates the positivity threshold of each biomarker, which was defined as 2 SD above the mean of the CU- group.


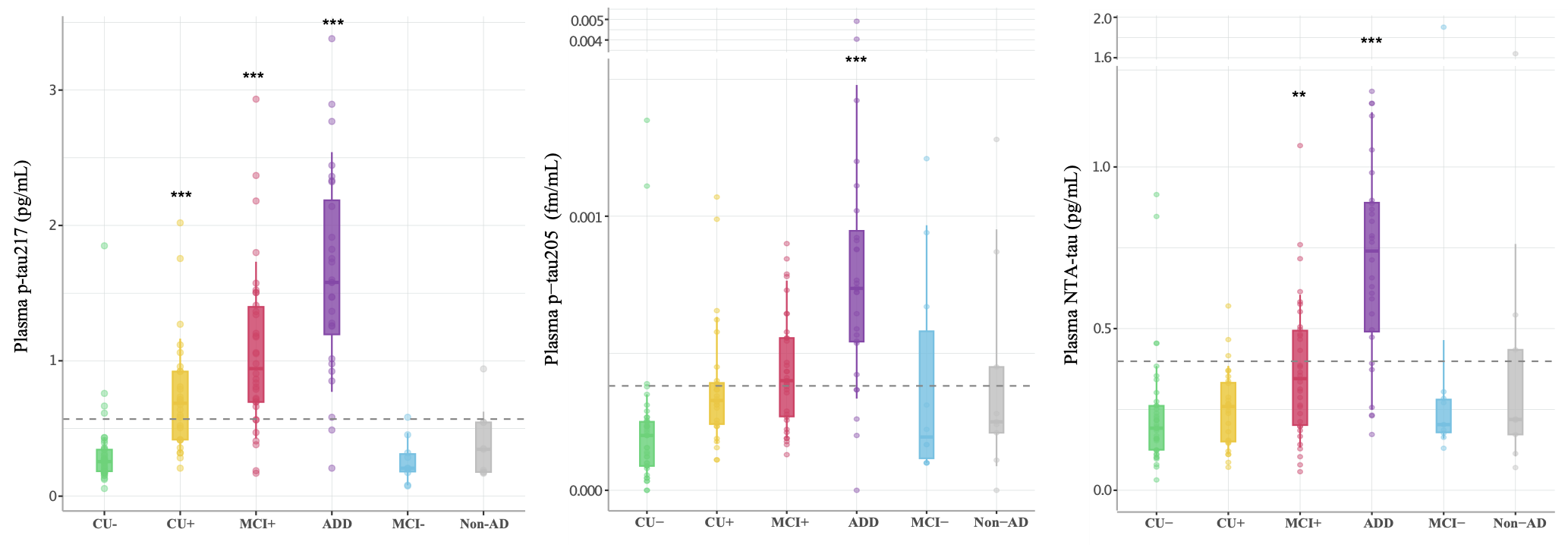


**Figure S2.** **Plasma tau biomarkers across disease groups.** The boxplots depict the median (horizontal bar), 25th to 75th percentiles (hinges) and whiskers indicate 10th and 90th percentiles. Group comparisons were computed with linear regression models adjusting for age and sex. The * indicates, for each biomarker, the groups that are significantly different from CU- group (**P*<0.05, ***P*<0.01,

****P*<0.001). The dashed line indicates the positivity threshold of each biomarker, which was defined as 2 SD above the mean of the CU- group.

**

**

**Figure S3.** **Scatterplots showing the distribution of CSF and plasma tau biomarkers across fluid stages.** Cut‐off values are displayed with black dashed lines, resulting in four quadrants, each of them representing the four different positive or negatively status for each biomarker pair. Participant color coding is based on fluid biomarker stage. Biomarker concentrations are log-transformed to facilitate visualization.

**Figure S4.** **CSF tau biomarkers across tau PET load.** The scatterplots depict CSF biomarker values (log2 scale) in relation to Tau PET SUVR in the medial temporal (top) and neocortical (bottom) regions. The line represents the locally estimated scatterplot smoothing (LOESS) regression.

**Figure S5. Tau-PET tracer uptake across CSF and plasma stages in different Braak regions (upaired samples).** The boxplots depict the median (horizontal bar), 25th to 75th percentiles (hinges) and whiskers indicate 10th and 90th percentiles. Linear regression models contrasted a higher stage to its preceding stage, adjusting for age and sex. The * indicates significantly different tau-PET uptake.

**Figure S6. Regional Aβ-PET uptake across CSF and plasma stages (unpaired samples).** Average Aβ-PET uptake across the brain for each of the **(A)** CSF-based stages and **(B)** plasma-based stages. The boxplots depict the median (horizontal bar), 25th to 75th percentiles (hinges) and whiskers indicate 10th and 90th percentiles. Linear regression models contrasted a higher stage to its preceding stage, adjusting for age and sex. The * indicates significantly different global Aβ-PET SUVR uptake in **(C)** CSF- and **(D)** plasma-based stages.

**

**

**Figure S7. Fluid-based staging across clinically relevant stratifications in paired CSF and plasma samples.** Stacked bar charts represent the percentages of fluid stage-0, stage-1, stage-2 and stage-3 participants. In blue, CSF staging across (**A**) diagnostic groups, (**B**) AT groups, (**C**) tau-PET defined Braak stages, and (**D**) tau-PET status. In pink, plasma staging across (**E**) diagnostic groups, (**F**) AT groups, (**G**) tau-PET defined Braak stages, and (**H**) tau-PET status. Discordant cases with the staging criteria are shown in grey. Table below shows the percentages and total number of individuals in each group.

**Figure S8.** Tau-PET tracer uptake across CSF and plasma stages in different Braak regions (paired samples).
